# Global, regional, and national burden and trend of diabetes in 195 countries and territories: an analysis from 1990 to 2025

**DOI:** 10.1038/s41598-020-71908-9

**Published:** 2020-09-08

**Authors:** Xiling Lin, Yufeng Xu, Xiaowen Pan, Jingya Xu, Yue Ding, Xue Sun, Xiaoxiao Song, Yuezhong Ren, Peng-Fei Shan

**Affiliations:** 1 Department of Endocrinology and Metabolism, The Second Affiliated Hospital of ZheJiang University School of Medicine, 88 Jiefang Road, Hangzhou, 310009 Zhejiang China; 2 Department of Ophthalmology, The Second Affiliated Hospital of ZheJiang University School of Medicine, 88 Jiefang Road, Hangzhou, 310009 Zhejiang China; 3 Department of General Practice, The Second Affiliated Hospital of ZheJiang University School of Medicine, 88 Jiefang Road, Hangzhou, 310009 Zhejiang China

**Keywords:** Diabetes, Risk factors

## Abstract

Diabetes mellitus is a leading cause of mortality and reduced life expectancy. We aim to estimate the burden of diabetes by type, year, regions, and socioeconomic status in 195 countries and territories over the past 28 years, which provide information to achieve the goal of World Health Organization Global Action Plan for the Prevention and Control of Noncommunicable Diseases in 2025. Data were obtained from the Global Burden of Disease Study 2017. Overall, the global burden of diabetes had increased significantly since 1990. Both the trend and magnitude of diabetes related diseases burden varied substantially across regions and countries. In 2017, global incidence, prevalence, death, and disability-adjusted life-years (DALYs) associated with diabetes were 22.9 million, 476.0 million, 1.37 million, and 67.9 million, with a projection to 26.6 million, 570.9 million, 1.59 million, and 79.3 million in 2025, respectively. The trend of global type 2 diabetes burden was similar to that of total diabetes (including type 1 diabetes and type 2 diabetes), while global age-standardized rate of mortality and DALYs for type 1 diabetes declined. Globally, metabolic risks (high BMI) and behavioral factors (inappropriate diet, smoking, and low physical activity) contributed the most attributable death and DALYs of diabetes. These estimations could be useful in policy-making, priority setting, and resource allocation in diabetes prevention and treatment.

## Introduction

Diabetes is one of the largest global public health concerns, imposing a heavy global burden on public health as well as socio-economic development. Although incidence has started to decrease in some countries, the prevalence of diabetes has increased in recent decades in most other developed and developing countries^1–3^. To date, the International Diabetes Federation (IDF) have estimated that 451 million adults live with diabetes worldwide in 2017 with a projected increase to 693 million by 2,045 if no effective prevention methods are adopted^4^. The prevalence of both type 1 and type 2 diabetes among children and adolescents has also increased, and the estimates of children and adolescents below age 20 with type 1 diabetes now exceed one million^5^.

Diabetes is one of the top 10 causes of death globally. Together with cardiovascular disease, cancer and respiratory disease, these conditions account for over 80% of all premature noncommunicable diseases (NCDs) deaths^6^. Individuals with diabetes have a 2–3 folds risk of all-cause mortality^7^. Presence of diabetes is associated with increased mortality from infections, cardiovascular disease, stroke, chronic kidney disease, chronic liver disease, and cancer^8,9^. In addition, although progress has been made in promoting population health and extending life expectancy, diabetes is the second biggest negative total effect on reducing global health adjusted life expectancy worldwide^10^.

Overall the global burden of diabetes has increased significantly in recent decades and will continue to soar in the next few decades. By using all available data from administrative hospital and medical claims records, cause of death records, the published and unpublished literature, the Global Burden of Disease study (GBD) is unique in its approach to generating estimates including incidence, prevalence, death, and disability-adjusted life-years (DALYs) for all regions^11^. DALYs, a summary measure of total health loss, were generated by summing years of life lost due to premature death and years lived with disability. These data allow GBD to document disease burden from diabetes in the most comprehensive way longitudinally and to provide the necessary information for priority setting and planning of health services. This study aims to explore the latest trend in global and regional-specific diabetic burden by type, year, socioeconomic status and its associated risk factors, thus help to achieve the goal of prevention and control of NCDs in 2025^12^.

## Methods

### Data sources

Data were collected from a set of possible sources, which include 21 possible Global Health Data Exchange data types ranging from scientific literature to survey data to epidemiological surveillance data^11^. The GBD study 2017 provides detailed epidemiologic estimates of more than 350 diseases and injuries in 195 countries and territories from 1990 to 2017^11^. The overall GBD 2017 methodologies and specific diabetes methodology have been described^11,13,14^.

Diabetes was defined by codes E10 to E14 according to the International Statistical Classification of Diseases, Tenth Revision (ICD-10), in the GBD Study 2017^11^. Interested diabetic data including incidence, prevalence, death, and DALYs and their uncertainty intervals (UI) were collected from Global Health Data Exchange^15^. Secondary analyses were performed by year, age, regions and socioeconomic status. (detail methodology were appended in the Supplementary Materials). Ethics approval and informed consent were not required for this study because of public accessibility to the data.

### Global and national socioeconomic status

Gross national income (GNI), a measure of the total domestic and foreign output, was calculated using the World Bank Atlas method. Countries were divided into 4 categories according to GNI in 2017^16^. Low income ≤ $995; Lower-Middle income $996 to $3,895; Upper-Middle income $3,896 to $12,055; High income ≥ $12,056. Socio-demographic Index (SDI), an indicator of a location’s socio-demographic development, was calculated on average income per person, educational attainment, and total fertility rate^6^. The SDI ranges from 0 to 1, with a higher value implying a higher level of socioeconomic development. 2017 National SDI data were obtained from the Global Health Data Exchange^15^: high SDI (> 0.81), high-middle SDI (0.70–0.81), middle SDI (0.61–0.69), low-middle SDI (0.46–0.60), and low SDI (< 0.46).

### Diabetic burden and risk factors

The calculations for the attributable burden of a given risk-outcome pair was explained in GBD 2017^13^, attributable DALYs or mortality were estimated as total DALYs or mortality for the outcome multiplied by the population attributable fraction (PAF). The PAF represents the proportion that the outcome would be reduced in a given population and time if there was exposure to the counterfactual level of the theoretical minimum risk exposure level^13^. 13 risks factors were obtained including metabolic (i.e., high BMI), environmental and occupational (i.e., ambient particulate matter pollution and household air pollution from solid fuels), behavioral (i.e., tobacco (smoking and secondhand smoking), dietary (diet low in whole grains, diet low in nuts and seeds, diet low in fruits, diet high in sugar-sweetened beverages, diet high in processed meat, diet high in red meat), alcohol use, low physical activity). As there were interactions between risk factors, all risk factors associated with DALYs may not be equal to the sum of each one.

### Method for forecasting diabetic burden beyond 2017

Auto-Regressive Integrated Moving Average (ARIMA) model was widely used to forecast in epidemiology^17–19^. We applied ARIMA to forecast the burden of diabetes from 2018 to 2025. Python was used to establish the ARIMA model to generate estimation with the best model of automatically selected P, D and Q (See the Supplementary Statistical Analysis Methods for details).

### Statistical analysis

Data were expressed as value with 95% uncertainty interval (UI). Age-standardized rates of incidence, prevalence, death, DALYs were expressed as number per 100,000 population. Comparisons of national age-standardized rates of incidence, prevalence, death and DALYs among five SDI-based countries groups were assessed using the Kruskal–Wallis test, followed by Dunn's multiple comparisons. Association of age-standardized rates of incidence, prevalence, death, DALYs with SDI were tested via Nonlinear regression analyses. All statistical analyses were conducted using Prism software (version 8; GraphPad). A *P*-value less than 0.05 was considered statistically significant.

## Results

### Global trend of diabetic burden from 1990 to 2017

**Figure 1:**
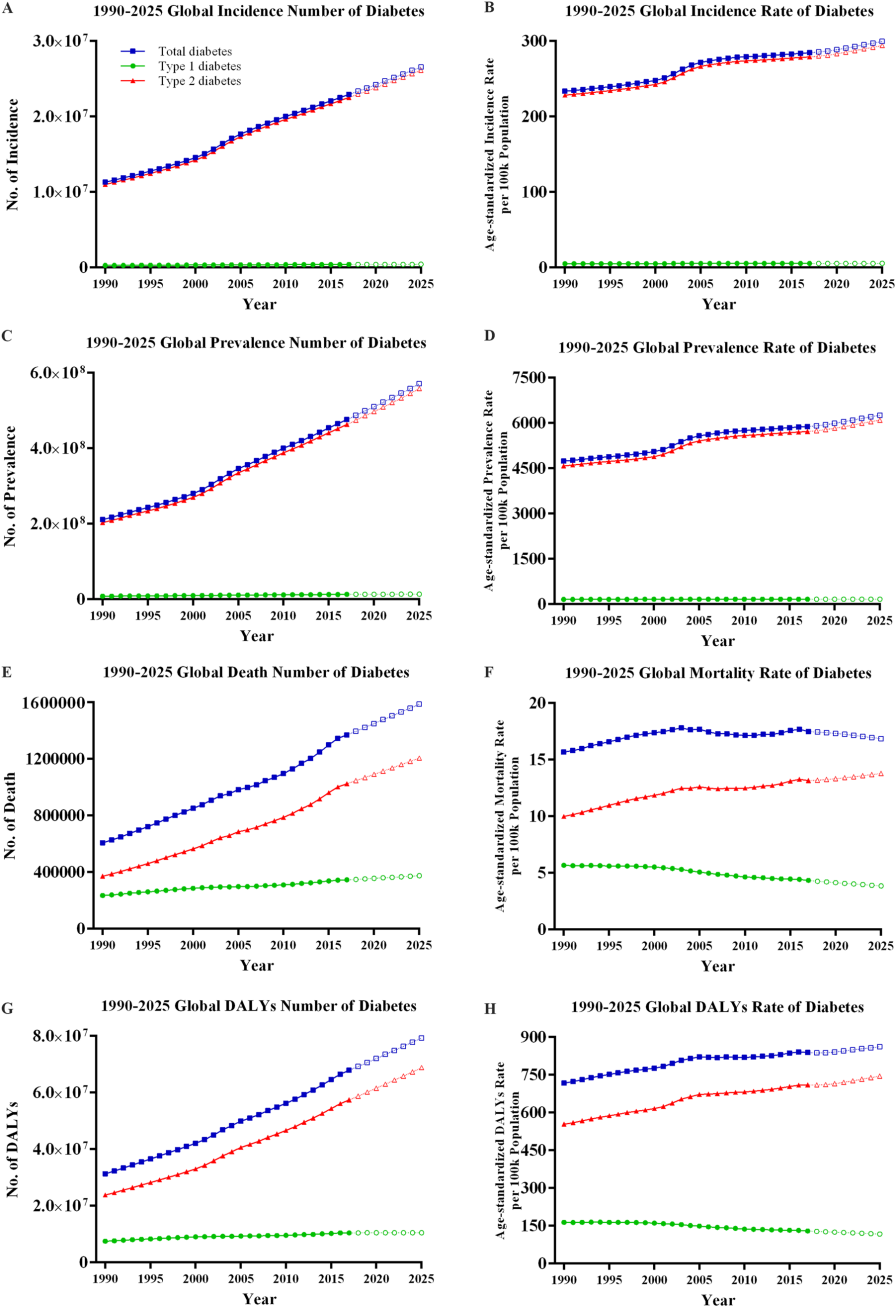
Global burden of diabetes mellitus from 1990 to 2025. (**A**) Incidence number; (**B**) Age-standardized incidence rate; (**C**) Prevalence number; (**D**) Age-standardized prevalence rate; (**E**) Death number; (**F**) Age-standardized mortality rate; (**G**) DALYs number; (**H**) Age-standardized DALYs rate. DALYs: disability-adjusted life-years.

The global disease burden of diabetes increased greatly from 1990 to 2017 (Fig. 1). Globally, the incidence of diabetes increased from 11.3 million (95% UI 10.6–12.1) in 1990 to 22.9 million (21.1–25.4) in 2017, with a 102.9% increase. The age-standardized incidence rate increased from 233.6 (218.4–249.4) to 284.6 (262.2–309.7). The global prevalence of diabetes increased from 211.2 million (196.0–228.5) in 1990 to 476.0 million (436.6–522.8) in 2017, with a 129.7% increase. The age-standardized prevalence rate increased from 4,738.5 (4,404.1–5,111.2) to 5,886.9 (5,403.6–6,458.5) (Fig. 1A–D).

Global deaths due to diabetes increased from 0.61 million (0.59–0.62) in 1990 to 1.37 million (1.34–1.40) in 2017, with a 125.5% increase. The age-standardized death rate increased from 15.7 (15.3–16.1) to 17.5 (17.1–17.9). Global DALYs increased form 31.3 million (26.1–37.8) in 1990 to 67.9 (55.4–82.6) in 2017, with a 116.7% increase in DALYs. Age-standardized DALY rates increased from 717.7 (599.2–863.6) to 839.0 (685.1–1,020.1) (Fig. 1E–H).

The global trend of type 2 diabetes, which accounted for majority of diabetes, was similar with that of total diabetes (Fig. 1). From 1990 to 2017, age-standardized rates of type 2 diabetes increased from 228.5 (213.7–244.3) to 279.1 (256.6–304.3) for incidence, from 4,576.7 (4,238.6–4,941.9) to 5,722.1 (5,238.2–6,291.0) for prevalence, from 10.0 (9.5–10.6) to 13.2 (12.7–13.7) for death, and from 553.6 (435.1–696.5) to 709.6 (557.2–888.3) for DALYs, respectively. In terms of type 1 diabetes from 1990 to 2017 (Fig. 1), the age-standardized rate increased slightly from 5.1 (4.6–5.6) to 5.4 (4.9–6.0) for incidence, and from 161.7 (146.1–180.7) to 164.8 (148.4–184.9) for prevalence, respectively. The age-standardized rate decreased from 5.7 (5.2–6.3) to 4.3 (4.0–4.7) for death, and from 164.0 (151.8–180.7) to 129.4 (121.3–137.6) for DALYs, respectively.

### Forecast of diabetic burden beyond 2017

It can be observed that the diabetic burden increased gradually from 1990 to 2017 and are predicted with a rise from 2018 to 2025 in terms of incidence, prevalence, death and DALYs (Fig. 1). More specifically, there was a forecast increased to 26.6 million, 570.9 million, 1.59 million, and 79.3 million in 2025 without effective interventions, respectively.

### Global burden of diabetes by super regions and countries

Generally, mortality and DALYs associated with diabetes experienced an increased trend in most GBD super regions (Table 1). The highest observed age-standardized mortality was in Oceania, followed by Sub-Saharan Africa, Southeast Asia, and Central Latin America regions. The highest age-standardized DALYs were mainly located in Oceania, Southeast Asia, Sub-Saharan Africa, Central Latin America, and the Caribbean. The lowest four age-standardized mortality and DALYs rate were observed in High Income regions, Central Europe, Eastern Europe and East Asia regions, remaining stable or showing a slight decrease.

**Table 1:** Age-Standardized rates of mortality and DALYs due to diabetes by GBD super regions in 1990, 2007, and 2017.

GBD 2017 super region	Mortality rate			DALYs rate		
	1990	2007	2017	1990	2007	2017
**High-income**						
High-income Asia Pacific	6.9 (6.8, 7.0)	5.3 (5.2, 5.4)	4.1 (3.9, 4.3)	457.2 (358.1, 577.2)	403.3 (308.6, 515.9)	410.0 (297.6, 538.2)
Western Europe	11.8 (11.7, 12.0)	9.4 (9.3, 9.6)	7.7 (7.4, 7.9)	494.0 (402.2, 606.6)	496.9 (386.8, 630.3)	514.1 (384.6, 668.0)
Australasia	10.3 (10.0, 10.5)	10.2 (10.0, 10.6)	8.1 (7.5, 8.8)	503.0 (405.8, 621.2)	444.1 (358.1, 547.4)	434.8 (336.3, 551.3)
High-income North America	13.4 (13.1, 13.6)	14.8 (14.6, 15.0)	12.1 (11.8, 12.5)	641.1 (534.0, 777.7)	770.2 (629.1, 939.9)	764.1 (607.5, 947.7)
Southern Latin America	16.7 (16.3, 17.1)	18.2 (17.8, 18.7)	16.1 (14.9, 17.4)	752.4 (623.9, 919.9)	810.0 (666.4, 996.5)	781.8 (627.4, 973.0)
**Eastern Europe/Central Asia**						
Central Europe	11.9 (11.7, 12.1)	11.6 (11.4, 11.8)	11.0 (10.7, 11.3)	665.3 (541.1, 819.4)	680.9 (539.4, 855.2)	694.8 (534.8, 883.5)
Eastern Europe	4.7 (4.6, 4.8)	4.6 (4.5, 4.6)	5.7 (5.6, 5.8)	509.6 (389.4, 657.5)	540.7 (408.7, 699.3)	537.8 (399.9, 693.3)
Central Asia	9.2 (9.0, 9.4)	18.2 (17.7, 18.8)	19.4 (18.3, 20.5)	775.5 (606.0, 984.8)	1,081.8 (879.4, 1,328.2)	1,130.8 (903.8, 1,398.1)
**Latin America and Caribbean**						
Tropical Latin America	27.8 (27.2, 28.4)	27.2 (26.6, 27.8)	26.2 (25.6, 26.8)	967.5 (841.6, 1,115.2)	912.8 (792.6, 1,053.4)	875.6 (751.6, 1,016.8)
Central Latin America	39.9 (39.3, 40.6)	37.8 (37.4, 38.3)	39.8 (38.6, 41.1)	1,524.9 (1,315.9, 1,768.4)	1,446 (1,233.4, 1,690.9)	1,567.6 (1,327.3, 1,860.8)
Andean Latin America	17.1 (16.0, 18.2)	20.2 (19.2, 21.3)	21.8 (20.1, 23.4)	739.1 (622.7, 889.7)	838.3 (708.6, 1,005.5)	896.2 (747.8, 1,080.0)
Caribbean	38.8 (37.1, 41.2)	34.9 (33.2, 37.0)	34.4 (32.5, 36.7)	1,442.9 (1,250.9, 1,688.1)	1,364.1 (1,166.1, 1,611.9)	1,382.5 (1,176.7, 1,640.5)
**Southeast/East Asia and Oceania**						
Southeast Asia	33.7 (31.3, 36.2)	35.6 (34.4, 37.0)	37.1 (35.6, 38.7)	1,250.1 (1,094.3, 1,450.5)	1,355.9 (1,179.8, 1,579.0)	1,479.6 (1,264.7, 1,738.1)
East Asia	8.4 (8.0, 8.9)	8.9 (8.7, 9.1)	8.8 (8.5, 9.1)	511.0 (405.7, 641.4)	605.1 (465.6, 768.3)	533.8 (412.2, 682.7)
Oceania	65.8 (60.9, 73.9)	101.8 (94.8, 111.2)	98.6 (89.7, 108.4)	2,492.9 (2,165.1, 2,915.4)	3,501.4 (3,107.2, 4,033.1)	3,430.8 (2,987, 4,006.4)
**North Africa and Middle East**						
North Africa and Middle East	25.3 (23.6, 27.4)	23.4 (22.4, 24.3)	22.6 (21.3, 23.8)	1,025.1 (867.1, 1,219.3)	1,066.3 (880.3, 1,284.1)	1,102.0 (899.4, 1,357.4)
**South Asia**						
South Asia	19.1 (17.7, 20.7)	27.2 (25.5, 28.4)	29.7 (28.1, 31.2)	734.1 (620.7, 870.0)	954.8 (810.1, 1,128.9)	1,011.0 (859.8, 1,191.9)
**Sub-Saharan Africa**						
Southern Sub-Saharan Africa	35.4 (32.4, 38.5)	66.3 (64.0, 68.4)	56.4 (54.0, 59.2)	1,342.8 (1,141.1, 1,561.0)	2,132.8 (1,880.4, 2,432.3)	1,912.3 (1,642.1, 2,214.1)
Eastern Sub-Saharan Africa	46.0 (42.1, 49.7)	39.8 (37.2, 42.9)	36.4 (33.6, 39.6)	1,399.3 (1,232.9, 1,591.4)	1,279.6 (1,122.1, 1,468.5)	1,217.2 (1,040.5, 1,420.3)
Central Sub-Saharan Africa	44.0 (39.3, 48.6)	42.1 (36.8, 47.4)	40.2 (34.8, 45.7)	1,592.4 (1,361.2, 1,875.7)	1,631.1 (1,354.1, 1,934.1)	1,657.8 (1,351.8, 1,987.6)
Western Sub-Saharan Africa	19.5 (17.3, 22.2)	25.9 (23.0, 29.1)	25.7 (23.2, 28.8)	722.5 (613.4, 852.6)	918.9 (777.7, 1,084.9)	954.3 (799.9, 1,134.3)

Age-standardized rate of mortality and DALYs were expressed as number per 100,000 population. Data were expressed as value with 95% uncertainty interval (lower, upper).

**Figure 2:**
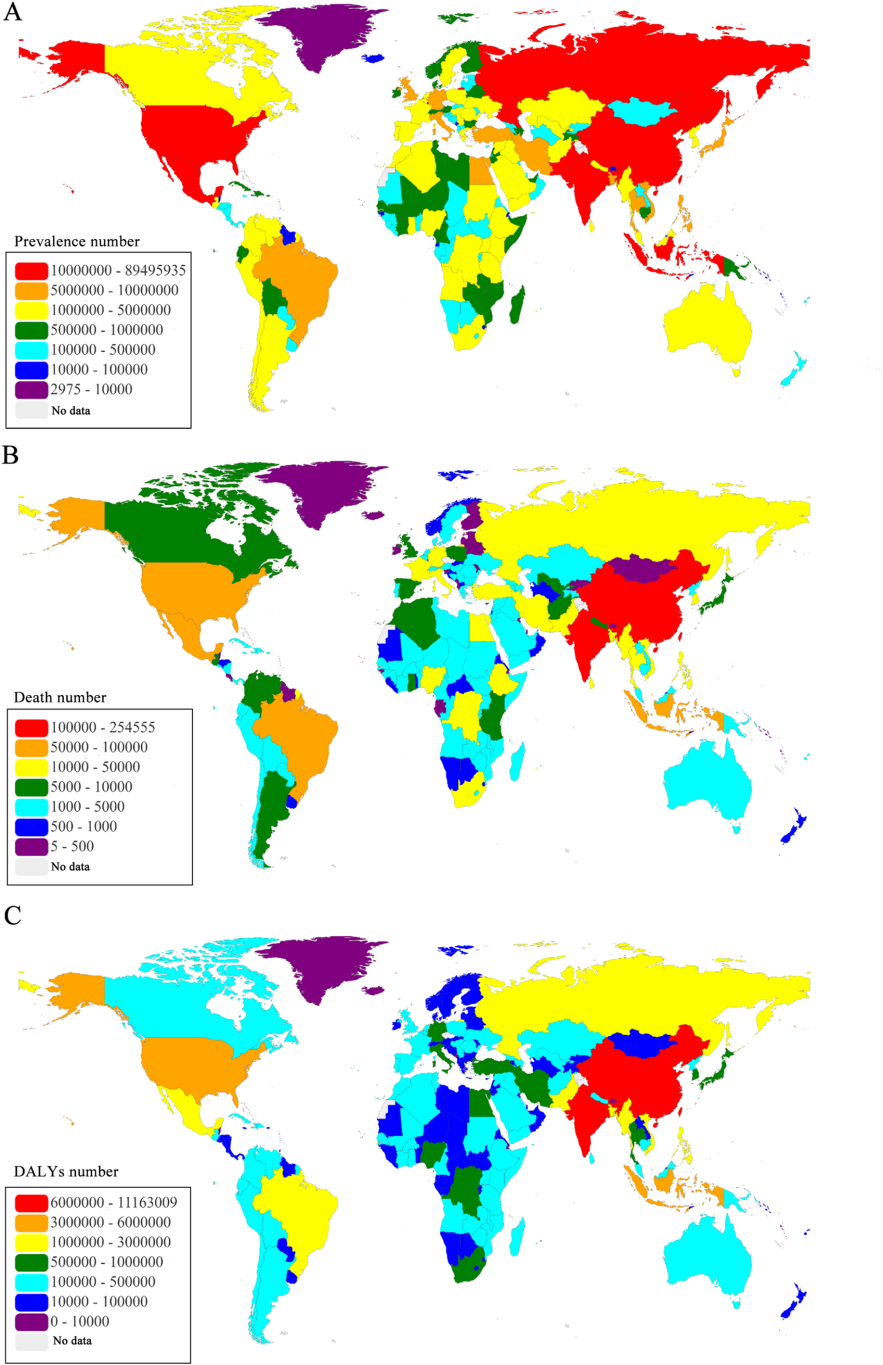
Global map of health burden of diabetes mellitus in 2017. (**A**) Prevalence number; (**B**) Death number; (**C**) DALYs number. DALYs: disability-adjusted life-years. Maps was based on EChart which is an open-source visualization library (under an Apache 2.0 license: https://github.com/apache/incubator-echarts/blob/master/LICENSE; URL of Echart: https://echarts.apache.org/zh/index.html), and implemented in Java (version JDK 1.8.0) using the software of IntelliJ IDEA Community Edition 2018.3.2.

The geographic distribution of diabetic burden in 2017 varied by countries (Fig. 2, Figure S1). The five highest prevalence were observed in China (89.5 million), India (67.8 million), United States (30.7 million), Indonesia (21.0 million), and Mexico (13.1 million). The top five countries of deaths were India (254,555), China (153,185), Indonesia (97,005), United States (68,558), and Mexico (64,067). The leading five countries of DALYs were India (11.2 million), China (10.0 million), Indonesia (4.4 million), United States (3.9 million), and Mexico (2.6 million). Detail distribution information of age-standardized prevalence, mortality and DALYs rates of diabetes were displayed in Figure S1.

### Global burden of diabetes by socioeconomic status

**Figure 3:**
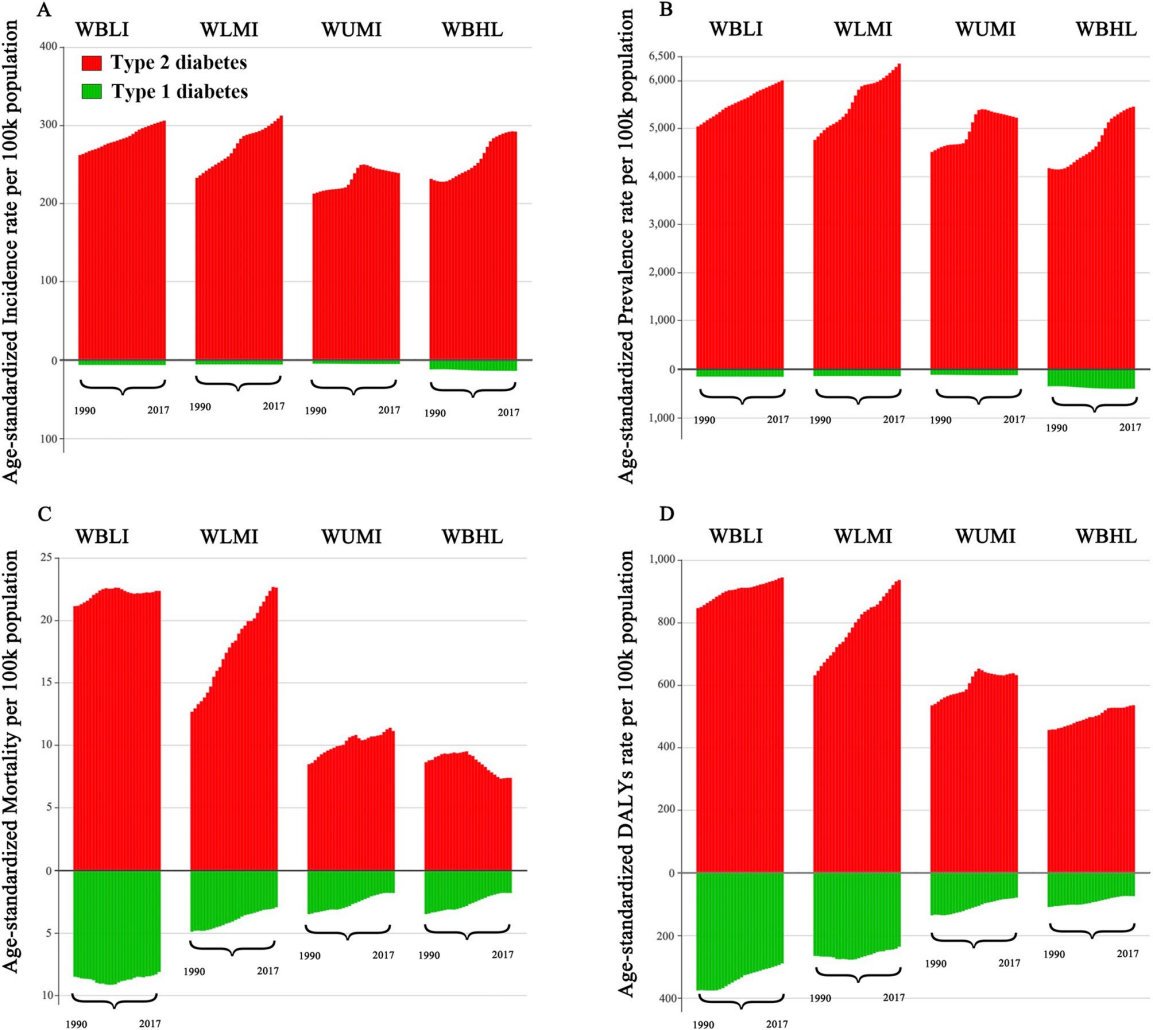
Global burden of diabetes mellitus in different World Bank Income Level regions from 1990 to 2017: (**A**) Age-standardized incidence rate, (**B**) Age-standardized prevalence rate, (**C**) Age-standardized mortality rate; (**D**) Age-standardized DALYs rate. WBLI: World Bank low income; WBLMI: World Bank lower middle income; WBUMI: World Bank upper middle income; WBHI: World Bank high income.

Based on the World Bank Income Level divisions, the incidence and prevalence of type 2 diabetes has increased greatly in all regions since 1990 (Fig. 3A,B). With exception of the high-income regions, the remaining regions had an increase in the age-standardized mortality of type 2 diabetes especially in the lower-middle-income regions (Fig. 3C). The age-standardized DALYs rate of type 2 diabetes exhibited an increasing trend in overall income level regions, noticeably in the lower-middle-income regions (Fig. 3D). With respect to type 1 diabetes, the incidence and prevalence slightly increased in recent years in high-income regions and remained stable in the remaining regions (Fig. 3A,B). The age-standardized mortality and DALYs rates of type 1 diabetes trended downward in all regions (Fig. 3C,D).

According to the classification of SDI, age-standardized incidence, prevalence, mortality and DALYs rates varied among five groups by diabetes type (Figure S2–S5). The highest age-standardized incidence and prevalence rates of type 1 diabetes were observed in high SDI regions (Figure S2, S3); while the aged-standardized rates of mortality and DALYs were negatively correlated with SDI (Figure S4, S5). For total diabetes and type 2 diabetes, the association between incidence, prevalence, mortality or DALYs rates with SDI index displayed an inverse U-shaped curve with the higher rates occurring in low-middle, middle, and high-middle SDI countries.

### Diabetic burden attributable to risk factors

Globally, the burden of diabetes is prominently associated with metabolic risks (i.e., high BMI) and behavioral factors (i.e., poor diet, smoking, and low physical activity). In 2017, the leading three risk factors were high BMI, dietary risks and ambient particulate matter pollution. High BMI was responsible for 30.8% of deaths and 45.8% of DALYs; dietary risk was responsible for 24.7% of deaths and 34.9% of DALYs; ambient particulate matter pollution was responsible for 13.4% of deaths and 15.4% of DALYs.

**Figure 4:**
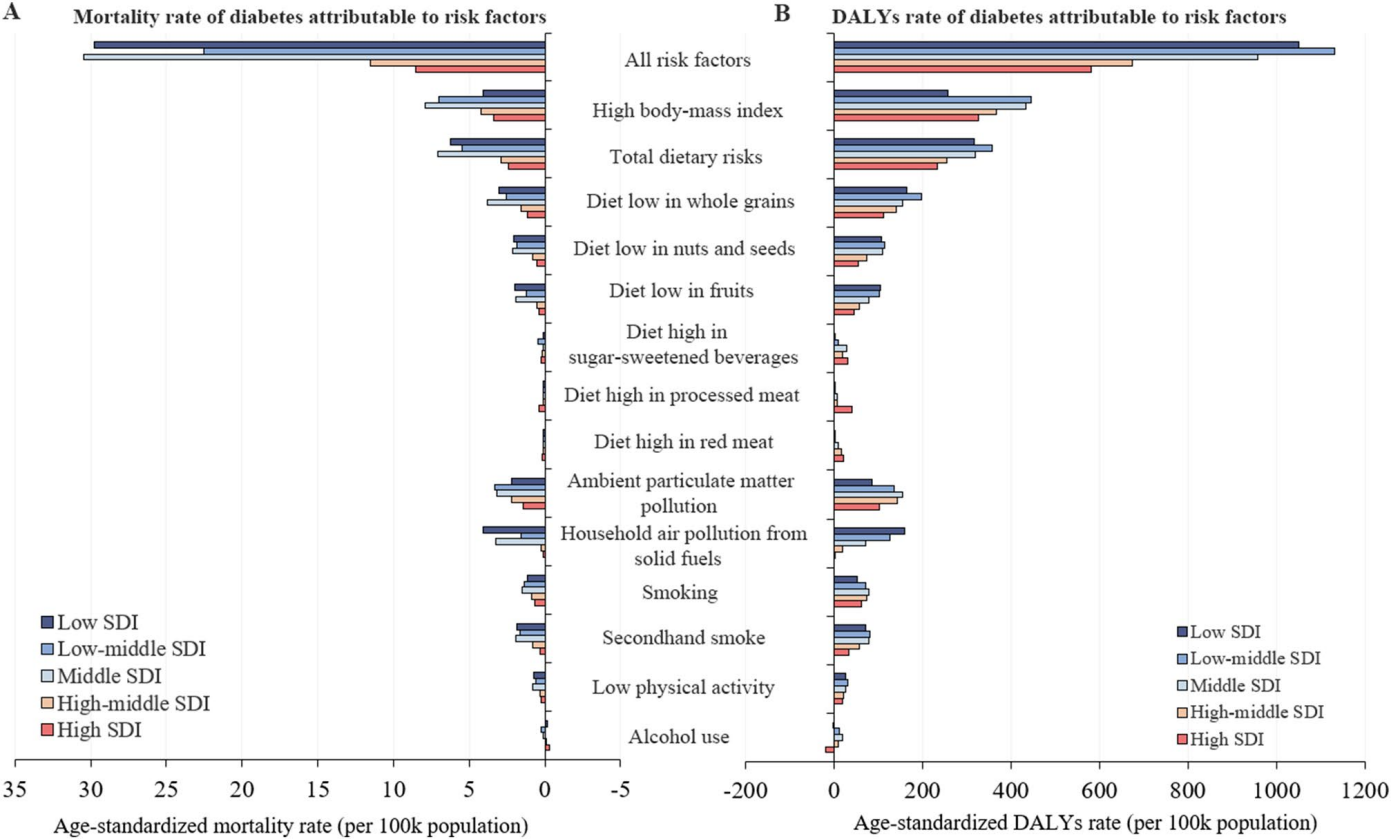
Age-standardized mortality rate and DALYs rate of diabetes attributable to risks by SDI level in 2017. (**A**) Age-standardized mortality rate; (**B**) Age-standardized DALYs rate. SDI: Socio-demographic Index.

The highest three burden of diabetes attributable to all risk factors, measured by age-standardized mortality and DALYs rate was observed in low-middle SDI, low SDI and middle SDI regions (Fig. 4). In low and low-middle SDI regions, diet low in fruits was a major risk factor. Moreover, low, low-middle, and middle SDI regions were places where there was greater risk of deaths and DALYs from household air pollution from solid fuels. Interestingly, alcohol use appeared to be a protective effect in high-SDI regions.

## Discussion

This study presented a comprehensive picture of the numbers, rates, and increased trends of the burden of diabetes in 195 countries and territories over the past 28 years. In 2017, the global prevalence number and DALYs number of diabetes reached 476.0 million and 67.9 million, with a 129.7% and 116.7% increase, respectively. There was a projected increased to 26.6 million of incidence, 570.9 million of prevalence, 1.59 million of death, and 79.3 million of DALYs in 2025 without effective interventions. Similarly, the global trend of type 2 diabetes also increased. However, the age-standardized rate of mortality and DALYs decreased steadily for type 1 diabetes. Interestingly, the low-middle, middle, and high-middle SDI regions were associated with higher burden, while high SDI regions had lower burden. In addition, modifiable metabolic, environmental, and behavioral factors appeared to be most relevant risk factors for the diabetes burden.

These estimates of GBD demonstrated the large and inexorably increasing burden of diabetes in the world. The estimated global prevalence number of diabetes had almost doubled since 1990. The estimated number was similar to data from IDF, which reported 451 million people aged 18–99 years living with diabetes^4^. The prevalence number for subjects aged 1–19 years was also included in GBD data, which increased from 5.7 million in 1990 to 8.8 million in 2017. In addition, this study also showed that the increasing trend of global burden varied by diabetic type and regions. The incidence and prevalence number of type 2 diabetes increased worldwide, with higher incidence and prevalence in low-middle, middle, and high-middle SDI countries. The increasing trend of type 1 diabetes has principally occurred in high income regions including Europe and the United States, in which there has been a reported 2.7–4.0% annual increase in type 1 diabetes^1,20^. This indicates that people in low-middle, middle, and high-middle SDI countries could more be prone to type 2 diabetes because of social and economic transformation with increased food supply, a westernized diet and reduced physical activity. Effective interventions should be conducted to change unhealthy lifestyles. On the other hand, for patients with type 2 diabetes, some methods including short duration intensive insulin therapy and reduction of body weight by intensive lifestyle interventions could be adopted to reverse diabetes^21,22^.

This study demonstrated that the estimated death number due to diabetes has increased in recent decades and reached 1.37 million in 2017. However, the mortality rate of type 2 diabetes in high-income areas and the global mortality rate of type 1 diabetes showed a downward trend. This estimated death number is similar to the data reported by the World Health Organization (1.6 million in 2016)^23^ but significantly lower than that reported by IDF (5 million in 2017)^4^. The disparity in global death number between GBD and IDF most likely results from the data resources and methodology they used. GBD data come from death certificates listing diabetes as the condition most likely to be associated with direct mortality. In contrast, the mortality reported by IDF is calculated from relative risks and total numbers of deaths from cohort studies comparing death rates in those with and without diabetes including both direct and indirect mortality^4^. It is well known that diabetes is associated with increasing incidence and death due to cardiovascular and cerebrovascular disease, cancer, infectious disease, which results in increased indirect mortality associated with diabetes^24^.

Our estimates showed that there was more than 67 million DALYs of diabetes in 2017. Age-standardized DALYs rates of type 2 diabetes exhibited an upward trend while type 1 diabetes showed a downward trend from 1990 to 2017. Globally, there were 706 million DALYs due to NCDs in 2017 and diabetes was one of the five leading NCDs causes of absolute risk-attributable DALYs^11^. Several previous studies have reported that diabetic DALYs showed an increasing trend in developing countries. In India, diabetes had the highest increase in DALYs rate among the NCDs from 1990 to 2016, with an age-standardized increase of 39.6%^25^. In China, DALYs increased by 95% from 1990 to 2016 and age-standardized DALYs rates increased by 2.3%^26^. In the Eastern Mediterranean Region, total DALYs increased by 191.3% from 1990 to 2015^27^. In contrast, there has been a decrease in DALYs in some developed countries. In the UK from 1990 to 2010, the DALYs number declined from 242,000 to 208,000, while the age-standardized DALYs rate decreased from 422 to 337^28^. Prevention and comprehensive control of diabetes including blood glucose, blood pressure and lipid profile should be emphasized, as these have all been shown to greatly decrease all-cause death and diabetic complications^29^.

Favorable changes occurred in the age-standardized mortality and DALYs rates in type 1 diabetes over the past 28 years, which is consistent with other studies. There is convincing evidence that individuals with type 1 diabetes have continuously improved since 1940s, with respect to living with their condition^30,31^. This could be closely related to the progress in diabetes education, continuous monitoring of blood sugar, the widespread use of insulin and insulin analogues, and sensor-augmented pump therapy^32^. In contrast, there are still some improvements that could be made when caring for those with type 1 diabetes, especially for low income and low-middle income countries, which presented lower prevalence but higher rates of mortality and DALYs. There is also a tremendous gap in life expectancy between patients with type 1 diabetes and that of the general population, even in high income countries. Recent studies demonstrated type 1 diabetes resulted in a loss of 10.2–17.7 life-years in Sweden, Scotland, and Taiwan region^31,33,34^. The situation is even worse for low income regions and low SDI regions where insulin is not readily available^35^. Strategies to balance distribution of medical and health resources in the world and inner a country should be formulated.

The higher increase in incidence and prevalence rates occurred in the lower-income regions as well as mortality and DALYs rate over the decades. This raises concerns for the less developed countries. When SDI was linked with DALYs, it revealed that type 1 diabetes DALYs were negatively associated with socioeconomic status; interestingly higher DALYs were observed in low-middle, middle, and high-middle SDI countries for total diabetes and type 2 diabetes. These highlight that type 1 diabetes is still severe due to limited access to essential medicines (particularly life-saving insulin) and technologies^35^. Multiple factors are involved in type 2 diabetes including biological, environmental, behavioral and social factors which make it more complicated to measure the association between the DALYs and SDI^36^. As low-middle, middle, and high-middle SDI countries are experiencing rapid socioeconomic progress, the dietary pattern and lifestyles change greatly. However, the basic infrastructures are insufficient to support healthy lifestyles and current healthcare services are unable to detect diabetic disease early and interfere timely. Intensive measures should be planned and implemented in the less developed countries to prevent further increases in diabetes DALYs and to reinforce healthcare services.

Our results showed that most of the diabetic burden is attributable to modifiable risk factors. High BMI contributed mostly to diabetes, with a general increase in DALYs rate (80.4%) and mortality rate (73.5%) since 1990. The high BMI risk in low-middle and middle SDI countries were significantly more severe. As is well reported, high BMI accounts greatly for diabetes and has been continuously rising^25^. The incidence of diabetes could be reduced by weight loss^37,38^. Globally, diet low in whole grains, nuts and seeds, and fruits were the leading risks among dietary risks, which were much more pronounced in lower SDI countries. Most developing countries are switching from traditional diets to higher intake of carbohydrates, fats and sugars^39^. Globalization and emerging supermarkets increase access to processed, high-fat, added-sugar and salt-laden foods. Relative low price and high accessibility of energy-dense but low-nutrient food decrease the consumption of whole grains, fruits and vegetables^39^. High BMI is greatly affected by dietary and physical activities which suggests policies could be targeted on improvements in healthy diet and adequate exercises. In developing countries, government can provide more incentives for purchasing whole grain, nuts and seeds, fruits and vegetables and put restrictions or disincentives for less healthy products. The age-standardized DALYs rate attributable to ambient particulate matter pollution experienced a 65.4% increase over the past 28 years. Though global household air pollution from solid fuels decreased, it was still a major risk factor in low and low-middle SDI countries. Ambient and household air pollution may alter lung function, vascular homeostasis and insulin sensitivity, resulting in abnormalities in glucose homeostasis^40^. Strengthening air pollution management is crucially needed. Climate change legislation and targets should help to improve air quality in industrialized countries.

The strengths of our study were the comprehensive estimations of diabetic burden as measured with prevalence, incidence, diabetes related death, and DALYs from 1990 to 2017. However, there were several limitations of this study. Firstly, our research was subjected to the methodologic defect of the GBD 2017^13^. For example, the GBD data is calculated by a Bayesian meta-regression tool based on reported data where the availability and quality of original data, and the method of data processing may introduce bias. Secondly, we should take bias in classification of diabetes into account. It was conducted by estimating overall diabetes and type 1 diabetes, then subtraction produced the estimation of type 2 diabetes. These included surveys may not accurately distinguish between type 1 and type 2 diabetes as it often requires relatively complicated laboratory tests to assess pancreas function. Thirdly, the mortality estimated by GBD comes from the certificates listing diabetes which may underestimate the death attributable to diabetes. Cause-specific mortality rate reflects the direct death caused by diabetes. Indeed, diabetes is closely associated with increasing risks and death of cardiovascular and cerebrovascular diseases, cancer, infectious disease which could lead to a higher indirect mortality rate. In addition, it is challenging to compare the diabetic disease burden considering the huge difference in health care access, quality of care and data quality between countries, and the conclusion should be cautiously interpreted in a specific district.

In conclusion, this study demonstrated the persistently increasing global burden of diabetes and variety by diabetic type, region, and countries. Furthermore, it provides the necessary information for priority setting and planning of health services to achieve the World Health Organization Global Action Plan for the Prevention and Control of NCDs in 2025, one of nine global NCDs targets to be attainted in 2025 is a 25% relative reduction in premature mortality from NCDs^12^. Given that most of the diabetes burden is precipitated by modifiable risk factors, government and academic organization are urgently required to make policies, allocate medical resources and edit clinical guidelines for diabetes education to change unhealthy lifestyles, effectively control the proportion of those who are overweight and obesity, therefore the incidence of diabetes could be reduced. Air pollution is one of the leading risk factors associated with the burden of diabetes, and it is necessary to enact policies to reduce environmental pollution and use clean energy to reduce indoor pollution.

## Supplementary information

Supplementary Information.

## Data Availability

Data are available in a public, open access repository. See: https://ghdx.healthdata.org/gbd-results-tool.
