# Supplementary material for: Global, regional, and national burden and trend of diabetes in 195 countries and territories: an analysis from 1990 to 2025
Source: Sci Rep. 2020 Sep 8;10:14790. doi: 10.1038/s41598-020-71908-9 (PMC7478957; doi:10.1038/s41598-020-71908-9)
Supplement: Supplementary file 1 — Supplementary Information. [file 41598_2020_71908_MOESM1_ESM.docx]

**Global, Regional, and National Burden and Trend of Diabetes in 195 Countries** **and Territories: an analysis from 1990 to 2025**

Xiling Lin^1*^, Yufeng Xu^2*^, Xiaowen Pan^1*^, Jingya Xu^1^, Yue Ding^1^, Xue Sun^3^, Xiaoxiao Song^1^, Yuezhong Ren^1^, Peng-Fei Shan^1#^

1. Department of Endocrinology and Metabolism, the Second Affiliated Hospital of ZheJiang University School of Medicine, 88 Jiefang Road, Hangzhou, Zhejiang, 310009, China

2. Department of Ophthalmology, the Second Affiliated Hospital of ZheJiang University School of Medicine, 88 Jiefang Road, Hangzhou, Zhejiang, 310009, China

3. Department of General Practice, the Second Affiliated Hospital of ZheJiang University School of Medicine, 88 Jiefang Road, Hangzhou, Zhejiang, 310009, China

* These authors contributed equally to this work.

# Corresponding Author:

Peng-Fei Shan, Department of Endocrinology and Metabolism, the Second Affiliated Hospital of ZheJiang University School of Medicine, 88 Jiefang Road, Hangzhou, Zhejiang, 310009, China; Phone: +86-0571-87784623; E-mail: pengfeishan@zju.edu.cn

**Supplementary** **Statistical Analysis Methods**

1. **Data source**

The Global Burden of Disease (GBD) study provides epidemiological data (including incidence, prevalence, mortality and disability-adjusted life-years (DALYs) etc.) of 345 diseases and injuries and 84 risk factors by age, sex in 195 countries and territories ranging from 1990 to 2017. Data of GBD study were collected from a set of possible sources, which include 21 possible Global Health Data Exchange data types ranging from scientific literature to survey data to epidemiological surveillance data.^1,2,3^ An integrative Bayesian meta-regression method that estimates a generalized negative binomial model for all epidemiological data was used through DisMod-MR 2.1 to generate the epidemiological data of disease burden; detail overall GBD 2017 methodologies and specific diabetes methodology see the Reference 1-3.

All GBD data used in this study were obtained from the Global Health Data Exchange,^4^ including (1) global incidence, prevalence, death, DALYs numbers and age-standardized rates (per 100,000 population) from 1990 to 2017; (2) global age-specific incidence, prevalence, death, DALYs numbers and crude rates in 2017; (3) National incidence, prevalence, death, DALYs numbers and age-standardized rates in 2017; (4) World Bank income level (WBIL) regional age-standardized rates of incidence, prevalence, death, and DALYs from 1990 to 2017; (5) GBD super region gender-specific age-standardized death rate and DALYs rate in 1990, 2007, and 2017. Raw data were provided by the Institute for Health Metrics and Evaluation (Seattle, Washington).

1. **Measures of burden**

Measures of burden at the global, regional, and national levels included incidence, prevalence, death and DALYs due to diabetes. DALYs were generated by summing years of life lost due to premature death and years lived with disability. The years of life lost represent the years lost due to premature death caused by diabetes; the years live with disability represents the years live with diabetes.

All data are reported as number and age-standardized rates per 100,000 population, where age standardization was based on the population recorded in registration systems in a country. The GBD Collaborators quantified and propagated uncertainty into final estimates by calculating uncertainty interval for cause-specific estimation components based on 1000 draws from the posterior distribution of cause-specific incidence/prevalence/death/DALYs by age, sex, location and year. (Detail estimation of uncertainty are shown in appendix of Reference 1-3).

1. **Data process**

GraphPad Prism software was used to generate the pattern of diabetic burden by year, age and region. Auto-regressive Integrated Moving Average model (ARIMA) was used to forecast the diabetic burden in terms of incidence, prevalence, death and DALYs. Python (version 3.7.4) was used to establish ARIMA model. Maps was based on EChart which is an open-source visualization library (under an Apache 2.0 license: https://github.com/apache/incubator-echarts/blob/master/LICENSE ; URL of Echart: https://echarts.apache.org/zh/index.html), and implemented in Java (version JDK 1.8.0) using the software of IntelliJ IDEA Community Edition 2018.3.2. Statistical analyses were conducted using GraphPad Prism software (version 8).

1. **ARIMA model**

ARIMA model was widely used in time series analysis. ARIMA is specified by three main component parameters known as P, D and Q. P stands for autoregression, represents the number of lag observations in the model; D stands for integrated, represents the number of times input raw data are differenced, in order to make the model stationary; Q stands for moving average, represents the size of moving average window applied to lagged observations. When the data were non-stationarity, differencing step can be applied one or more times to eliminate the non-stationarity. Python was used to establish ARIMA model. First, analysis the data and compute the difference between consecutive data to choose the P which makes the data stationary. Loop the steps “p<data.size/10,q<data.size” to generate all combination of P and Q, choose the P and Q with the minimum Bayesian Information Criterion, then generate prediction. The code is as follows.

#coding=gbk

import matplotlib.pyplot as plt

import pandas as pd

from statsmodels.tsa.arima_model import ARIMA

from statsmodels.graphics.tsaplots import plot_acf, plot_pacf

filename = r'death+rate+data .csv'

data = pd.read_csv(filename,usecols=['Year','Male'] ,index_col = u'Year',dtype={'Male': float})

plt.rcParams['font.sans-serif'] = ['SimHei']

plt.rcParams['axes.unicode_minus'] = False

init_d= 1

D_data = data.diff(init_d).dropna()

D_data.columns = [u'Male']

pmax = int(len(D_data) / 10)

qmax = int(len(D_data) / 10)

bic_matrix = []

for p in range(pmax +1):

temp= []

for q in range(qmax+1):

try:

temp.append(ARIMA(data, (p, init_d, q)).fit().bic)

except Exception:

temp.append(None)

bic_matrix.append(temp)

bic_matrix = pd.DataFrame(bic_matrix)

p,q = bic_matrix.stack().idxmin()

model = ARIMA(data, (p,init_d,q)).fit()

model.summary2()

forecast, fcasterr, conf_int = model.forecast(20)

for x in forecast:

print x

**References**

1. James, S. L. *et al.* Global, regional, and national incidence, prevalence, and years lived with disability for 354 Diseases and Injuries for 195 countries and territories, 1990-2017: A systematic analysis for the Global Burden of Disease Study 2017. *Lancet* **392**, 1789–1858 (2018).

2. Dicker, D. *et al.* Global, regional, and national age-sex-specific mortality and life expectancy, 1950-2017: A systematic analysis for the Global Burden of Disease Study 2017. *Lancet* **392**, 1684–1735 (2018).

3. Forouzanfar, M. H. *et al.* Global, regional, and national comparative risk assessment of 79 behavioural, environmental and occupational, and metabolic risks or clusters of risks, 1990–2015: a systematic analysis for the Global Burden of Disease Study 2015. *Lancet* **388**, 1659–1724 (2016).

4. Global Health Data Exchange. http://ghdx.healthdata.org/gbd-results-tool. *Institute for Health Metrics and Evaluation (IHME)* Accessed by 10 April 2019.


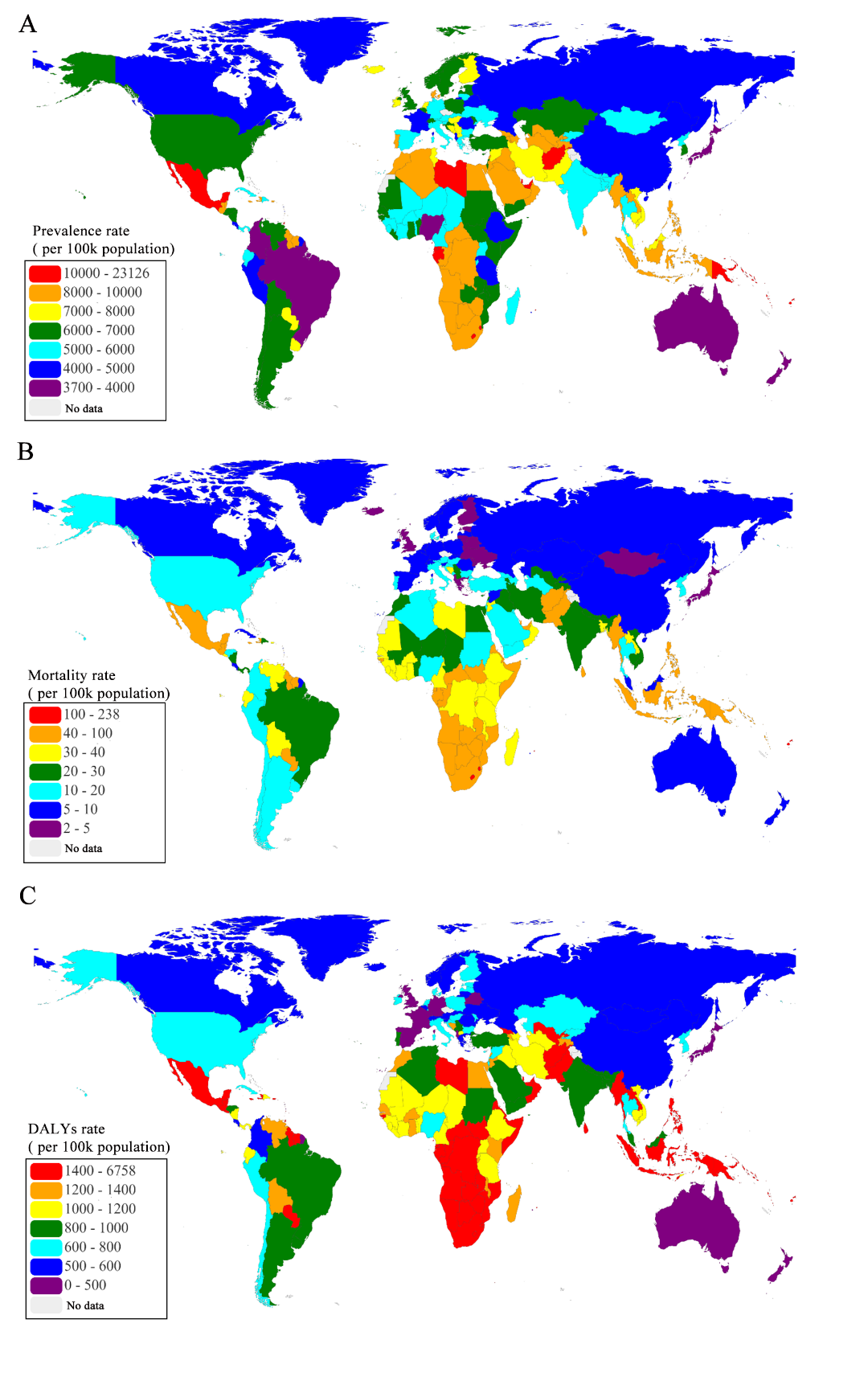


**Figure S1.** Global map of health burden of diabetes mellitus in 2017. (A) Age-standardized prevalence rate; (B) Age-standardized mortality rate; (C) Age-standardized DALYs rate. DALYs: disability-adjusted life-years. Maps was based on EChart which is an open-source visualization library (under an Apache 2.0 license: https://github.com/apache/incubator-echarts/blob/master/LICENSE ; URL of Echart: https://echarts.apache.org/zh/index.html), and implemented in Java (version JDK 1.8.0) using the software of IntelliJ IDEA Community Edition 2018.3.2.

**
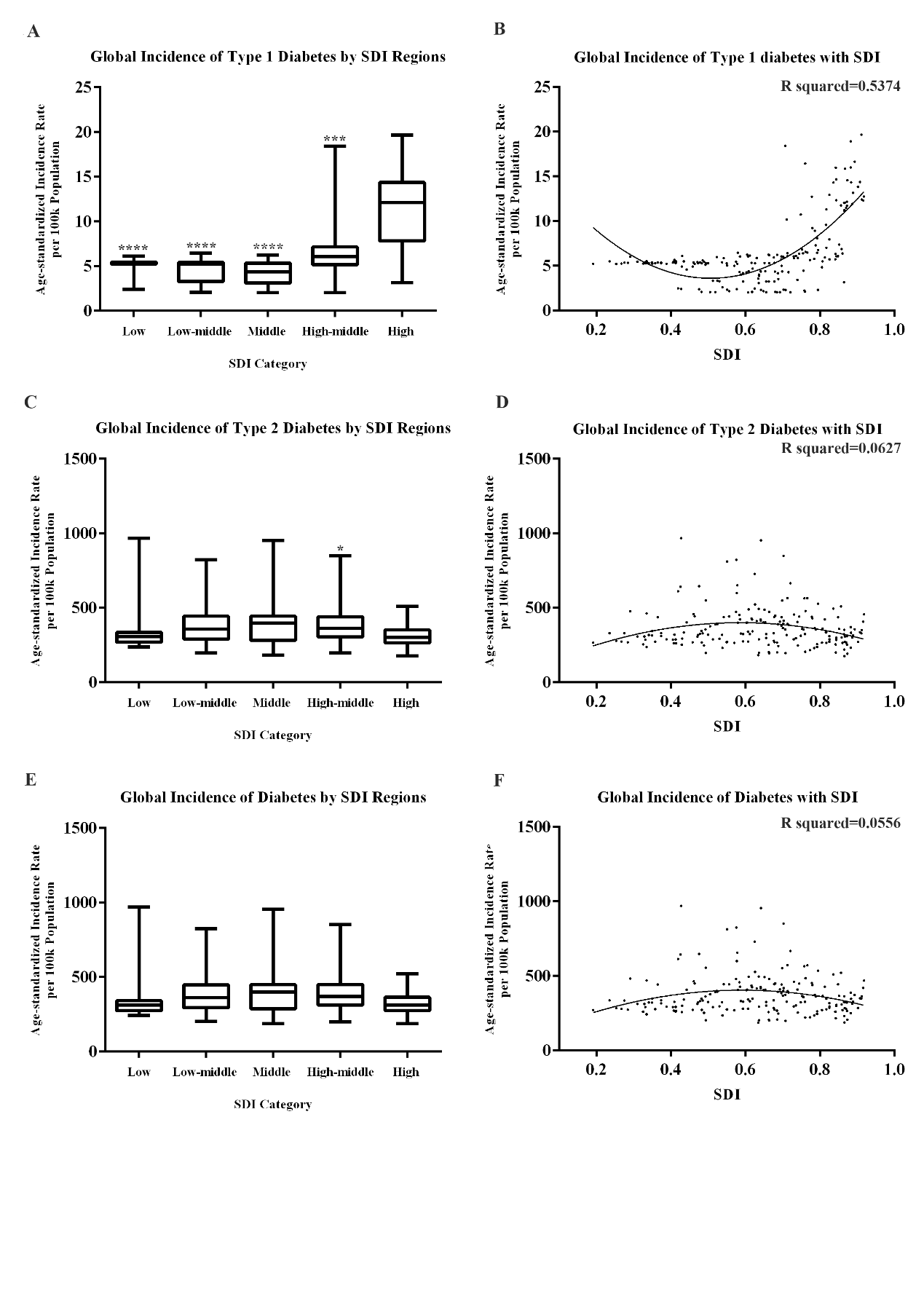
**

**Figure S2.** Global Burden of Diabetes Mellitus in SDI regions in 2017. (A) Age-standardized incidence of type 1 diabetes in different SDI regions; (B) Association between age-standardized incidence of type 1 diabetes with SDI; (C) Age-standardized incidence of type 2 diabetes in different SDI regions; (D) Association between age-standardized incidence of type 2 diabetes with SDI; (E) Age-standardized incidence of diabetes in different SDI regions; (F) Association between age-standardized incidence of diabetes with SDI. SDI: Socio-demographic Index. Every other group is compared with the high group. *: p< 0.05; ***: p< 0.001; ****: p< 0.0001.


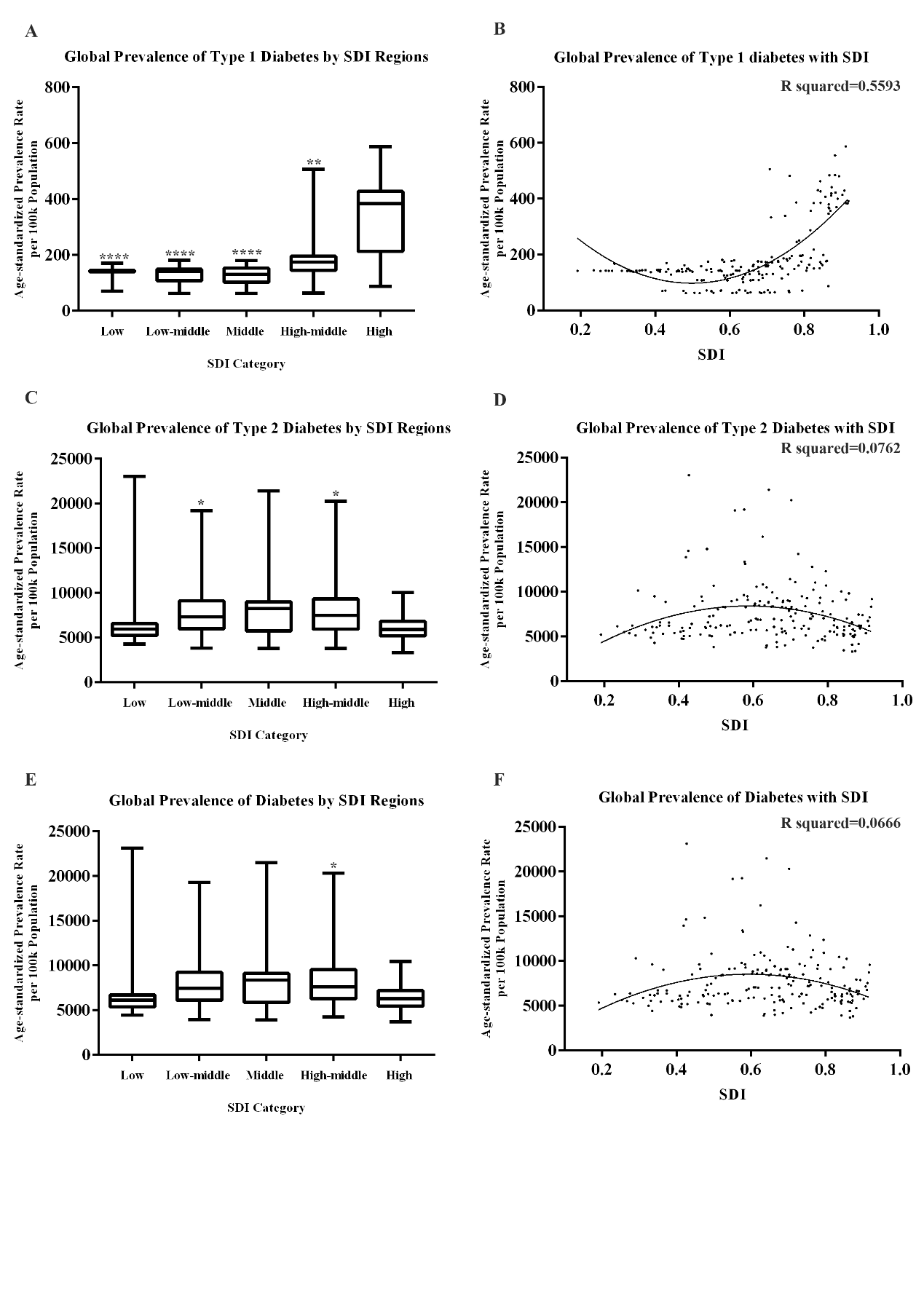


**Figure S3.** Global Burden of Diabetes Mellitus in SDI regions in 2017. (A) Age-standardized prevalence of type 1 diabetes in different SDI regions; (B) Association between age-standardized prevalence of type 1 diabetes with SDI; (C) Age-standardized prevalence of type 2 diabetes in different SDI regions; (D) Association between age-standardized prevalence of type 2 diabetes with SDI; (E) Age-standardized prevalence of diabetes in different SDI regions; (F) Association between age-standardized prevalence of diabetes with SDI. SDI: Socio-demographic Index. Every other group is compared with the high group. *: p< 0.05; **: p< 0.005; ****: p< 0.0001.


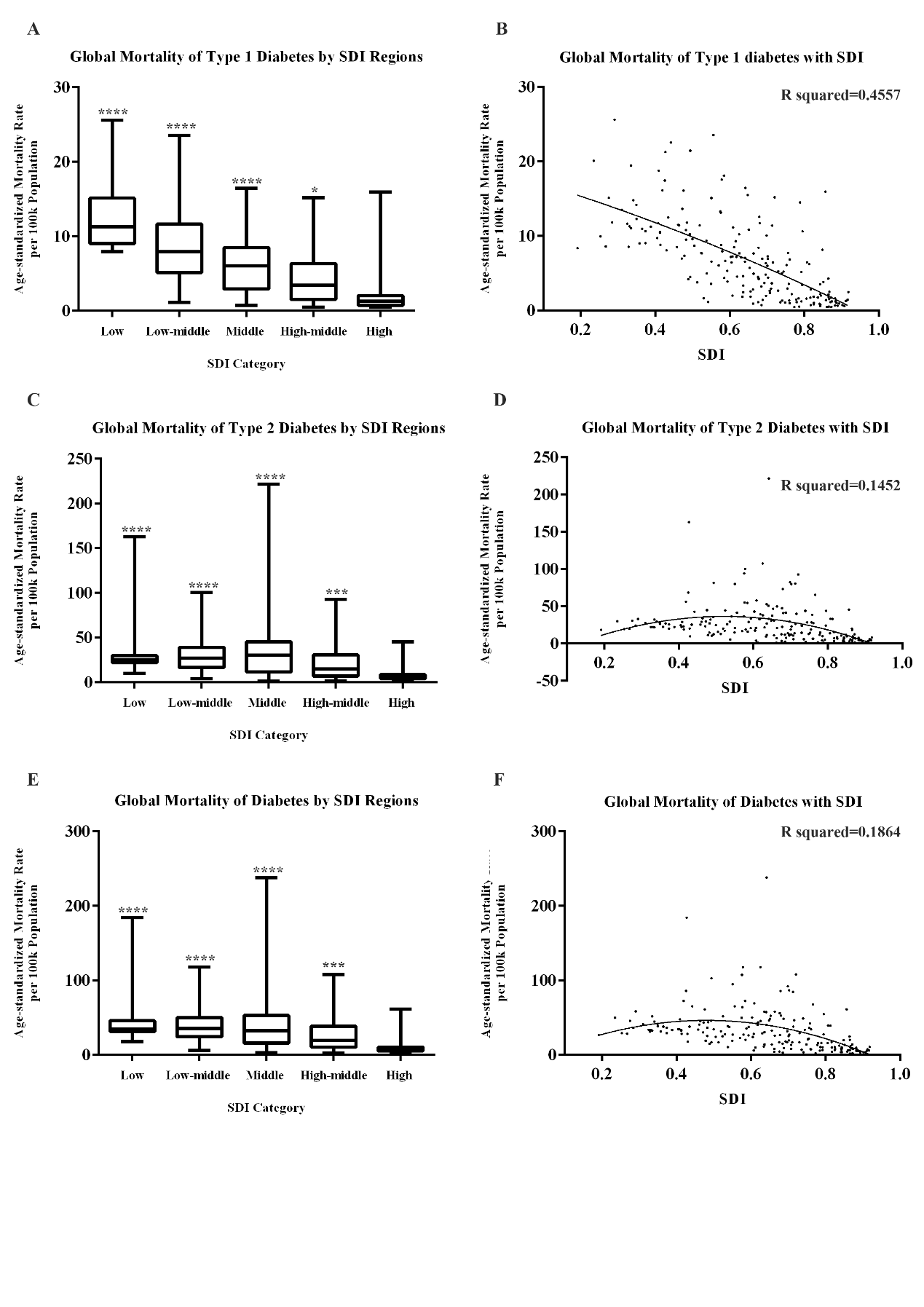
**Figure S4.** Global Burden of Diabetes Mellitus in SDI regions in 2017. (A) Age-standardized mortality of type 1 diabetes in different SDI regions; (B) Association between age-standardized mortality of type 1 diabetes with SDI; (C) Age-standardized mortality of type 2 diabetes in different SDI regions; (D) Association between age-standardized mortality of type 2 diabetes with SDI; (E) Age-standardized mortality of diabetes in different SDI regions; (F) Association between age-standardized mortality of diabetes with SDI. SDI: Socio-demographic Index. Every other group is compared with the high group. *: p< 0.05; ***: p< 0.001; ****: p< 0.0001.

**
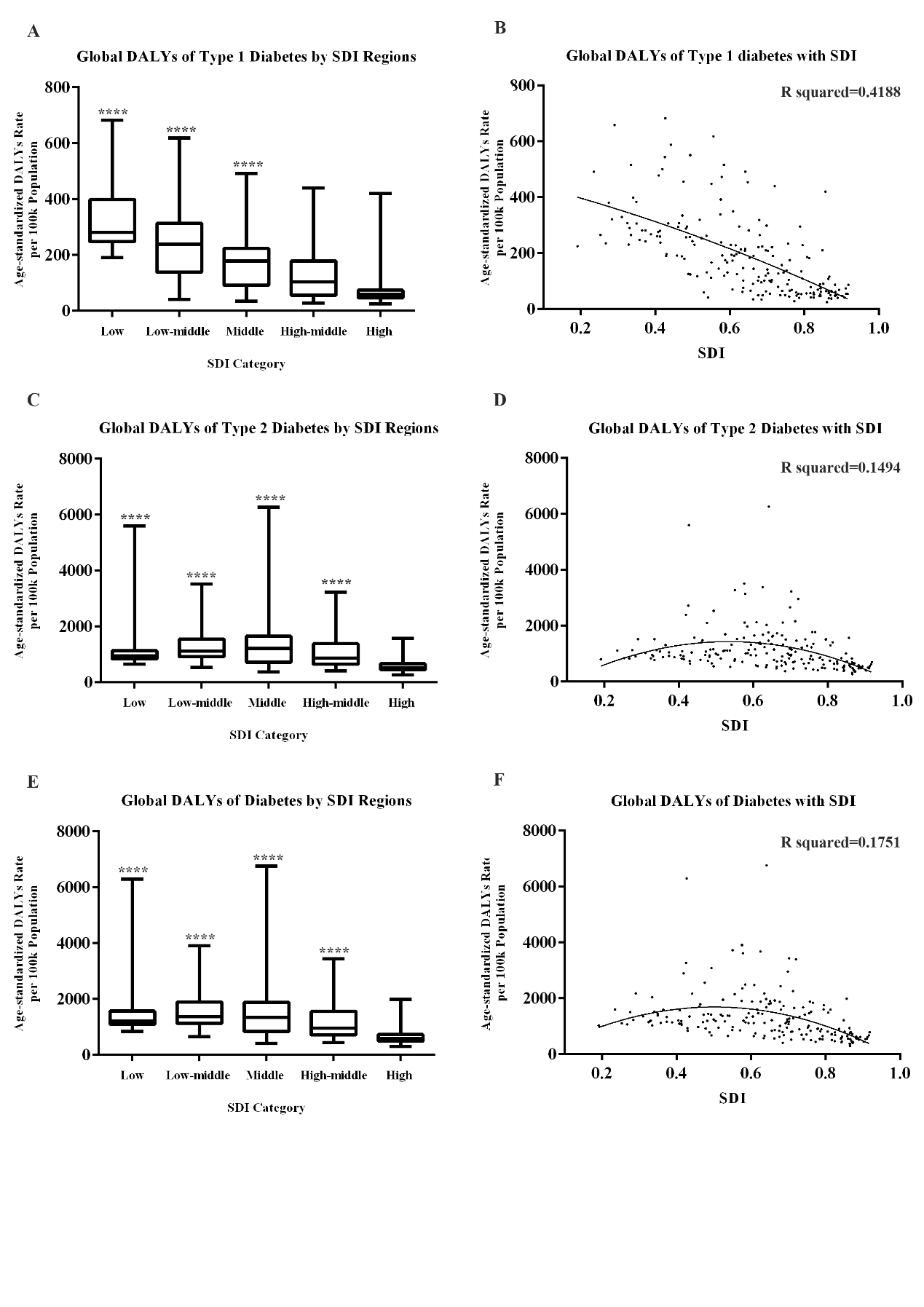
**

**Figure S5.** Global Burden of Diabetes Mellitus in SDI regions in 2017. (A) Age-standardized DALYs of type 1 diabetes in different SDI regions; (B) Association between age-standardized DALYs of type 1 diabetes with SDI; (C) Age-standardized DALYs of type 2 diabetes in different SDI regions; (D) Association between age-standardized DALYs of type 2 diabetes with SDI; (E) Age-standardized DALYs of diabetes in different SDI regions; (F) Association between age-standardized DALYs of diabetes with SDI. SDI: Socio-demographic Index. Every other group is compared with the high group. ****: p< 0.0001.
